# Onset hyperalgesia and offset analgesia: Transient increases or decreases of noxious thermal stimulus intensity robustly modulate subsequent perceived pain intensity

**DOI:** 10.1371/journal.pone.0231124

**Published:** 2020-12-08

**Authors:** Benedict J. Alter, Mya Sandi Aung, Irina A. Strigo, Howard L. Fields

**Affiliations:** 1 Department of Anesthesia and Perioperative Care, University of California San Francisco, San Francisco, California, United States of America; 2 Department of Anesthesiology and Perioperative Medicine, University of Pittsburgh, Pittsburgh, Pennsylvania, United States of America; 3 San Francisco VA Health Care System & Department of Psychiatry, University of California San Francisco, San Francisco, California, United States of America; 4 Department of Neurology, University of California San Francisco, San Francisco, California, United States of America; Global Neuroscience Initiative Foundation, UNITED STATES

## Abstract

Reported pain intensity depends not only on stimulus intensity but also on previously experienced pain. A painfully hot temperature applied to the skin evokes a lower subjective pain intensity if immediately preceded by a higher temperature, a phenomenon called offset analgesia. Previous work indicated that prior pain experience can also increase subsequent perceived pain intensity. Therefore, we examined whether a given noxious stimulus is experienced as more intense when it is preceded by an increase from a lower temperature. Using healthy volunteer subjects, we observed a disproportionate increase in pain intensity at a given stimulus intensity when this intensity is preceded by a rise from a lower intensity. This disproportionate increase is similar in magnitude to that of offset analgesia. We call this effect onset hyperalgesia. Control stimuli, in which a noxious temperature is held constant, demonstrate that onset hyperalgesia is distinct from receptor or central sensitization. The absolute magnitudes of offset analgesia and onset hyperalgesia correlate with each other but not with the noxious stimulus temperature. Finally, the magnitude of both offset analgesia and onset hyperalgesia depends on preceding temperature changes. Overall, this study demonstrates that the perceptual effect of a noxious thermal stimulus is influenced in a bidirectional manner depending upon both the intensity and direction of change of the immediately preceding thermal stimulus.

## Introduction

Controlled psychophysical studies using acute stimuli reveal a consistent relationship between stimulus intensity and reported subjective pain intensity [1]. This relationship reflects the activity of the peripheral and central nociceptive transmission pathways. However, both clinical and experimental studies have demonstrated that there can be a dramatic dissociation between subjective pain reports and the intensity of the applied nociceptive input. Reported pain is consistently and predictably modifiable by prior experience and expectation, such that equivalent noxious input can yield divergent pain reports when sensory cues associated with lower or higher pain are presented [2, 3]. Placebo analgesia and the increase in pain with nocebo are examples of this process [4, 5]. Importantly, there is a dynamic interaction between the intensity of ongoing pain perception and the impact of superimposed noxious stimulation [6]. Although much progress has been made in understanding how learned predictive cues influence subsequent pain perception, less is known about how temporal changes in nociceptive input can dynamically modify the perceptual impact of subsequent noxious input.

In an effort to better understand how nociceptive dynamics modulate pain perception, and specifically pain relief, Coghill and others made a significant advance when they discovered and provided detailed studies of what they labeled “offset analgesia” [7, 8]. Using a cutaneous thermode they found that when a one-degree Celsius increase in thermode temperature from a noxious target temperature is followed by a return to the target temperature there is a disproportionate drop in reported pain intensity rating compared to when the stimulus is held constant at the target temperature—so-called “offset analgesia”. This effect was also observed when the transient increase and decrease were applied on the contralateral arm consistent with a primarily central nervous system (CNS) mechanism for offset analgesia [9]. Functional imaging studies indicate that offset analgesia is mediated by brainstem pain modulatory circuitry [10–13]. Curiously, although these modulatory circuits are known to exert bidirectional control over nociceptive transmission, no disproportionately large increase in pain with the onset of the higher temperature was reported. Subsequently, elegant modeling of pain intensity with supra-threshold heat stimuli predicted perceptual enhancement of temperature increases as well as decreases [14], although this was not empirically demonstrated. Increases in radiant heat using infra-red light applied to the skin do elicit increases in pain intensity, but again these hyperalgesic changes were modest compared to offset analgesia [15].

To clarify the apparent contradiction between reports of offset analgesia without equivalent enhancement of temperature increases and the known bidirectional effects of descending modulation, we re-examined the effect of small changes of stimulus intensity on subsequent pain reports using a stimulus pattern that was similar to but the inverse of that used to elicit offset analgesia. In the current study, we report that robust onset hyperalgesia exists, consistent with bidirectional perceptual modulation dependent upon the direction of change of the immediately preceding noxious stimulus.

## Materials and methods

### Subjects

35 female and 39 male subjects signed written informed consent for the study, which was approved by the University of California, San Francisco (UCSF) Institutional Review Board. Subjects were recruited by public notice, including on-line advertising through Craigslist and paper advertising at UCSF. Inclusion criteria were healthy subjects aged 18–50 years old. Exclusion criteria included current significant medical comorbidities requiring frequent medical follow-up, pregnancy, chronic pain, ongoing acute pain, depression, anxiety, bipolar disorder, psychotic disorder, concurrent analgesic use, concurrent psychoactive medications (e.g. benzodiazepines, antidepressants), blindness, deafness, non-fluency in English. For consistency with ongoing studies, exclusion criteria also included inability to undergo magnetic resonance imaging (MRI) for any reason (e.g. non-compatible implants, claustrophobia).

### Testing protocol

The study timeline included recruitment and administration of standardized surveys followed by a single study visit lasting 2–3 hours. During the study visit, subjects completed surveys and then underwent heat pain threshold testing, heat stimulus calibration, and supra-threshold heat pain testing.

The study visit occurred in a research lab located in a clinical building on a hospital campus. A single office room was used. Experimenters included authors B.A. and S.A. All subjects were monitored using a 3-lead electrocardiogram (ECG) which was in place during testing. Breaks were allowed if the subject requested them. Subjects were compensated monetarily for their time and travel.

### Questionnaires

Upon study inclusion, subjects completed electronic (REDCap) or paper surveys. For most subjects, surveys were done electronically prior to study visit. If the electronic survey was not done or incomplete on the day of the study visit, a paper survey was done. The survey packet included self-reported basic demographic information, medical histories, and measures of social status (BSMSS), depression (BDI-II), anxiety (STAI Y-1 and Y-2), impulsivity (BIS-11) and pain catastrophizing (PCS). The BSMSS generates a single ordinal score reflecting the respondent’s education and occupation as well as the education and occupation of their parents and spouse [16]. The BDI-II measures cognitive-affective and somatic-vegetative aspects of depression with excellent psychometric properties across different populations [17]. The STAI measures both state (Y-1) and trait (Y-2) anxiety, producing an ordinal score reflecting apprehension, tension, nervousness, and arousal [18, 19]. The PCS measures catastrophic thinking associated with pain incorporating magnification of pain-related symptoms, rumination about pain, feelings of helplessness, and pessimism about pain-related outcomes [20]. The BIS-11 measures attentional, motor, and non-planning impulsiveness [21] which are associated with reward processing relevant to pain and addiction [22, 23]. After sensory testing outlined below, the STAI Y-2 and the situational pain catastrophizing scale (SPCS), measuring catastrophizing related to a pain experience [24], were administered.

### Equipment and pain reporting

**Fig 1:**
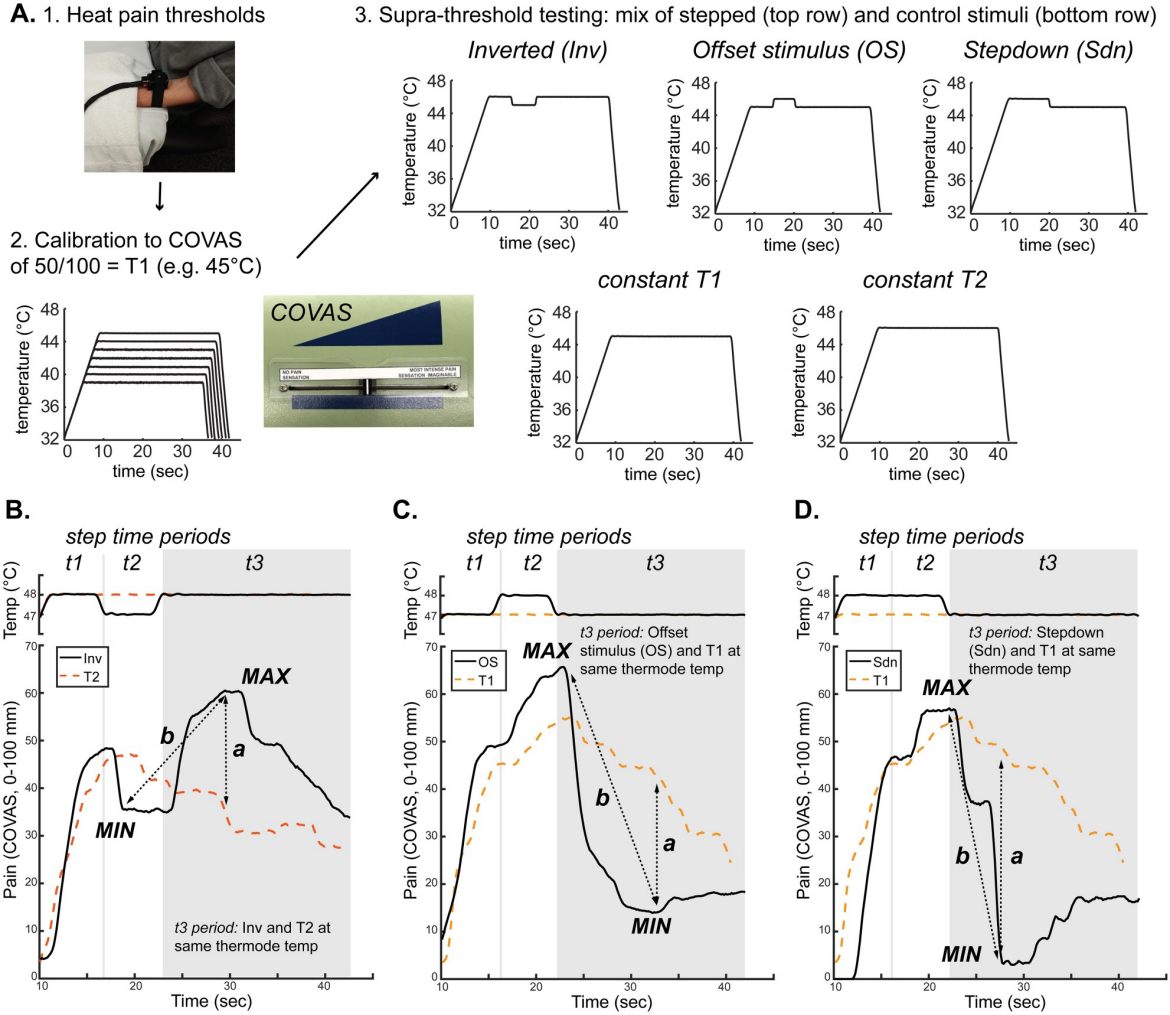
Experimental design and examples of data analysis. **A.** Subjects underwent (1) heat pain threshold testing, (2) an ascending series of suprathreshold, constant, 30-second, temperature stimuli to determine an individualized temperature that would elicit a COVAS pain rating of 50 mm/100 mm, and finally (3) a randomized mixture of suprathreshold, 30-second temperature stimuli testing the importance of direction of stimulus intensity change on pain perception. The mixture included transient, changing, supra-threshold noxious thermal stimuli (top row) and comparison constant stimuli (bottom row) shown. The data plotted are examples from a single subject in which T1 = 45°C. **B.–D.** Examples of continuous pain intensity measured by COVAS and thermode temperature during stepped stimuli with appropriate control stimuli superimposed from a different single subject in which T1 = 47°C. Inverted (Inv) stimulus data are plotted overlapping with the control T2 stimulus data to illustrate differences in subjective pain intensity produced by transient noxious thermal stimulus changes. During the t3 period (shaded area), both Inv and constant T2 stimuli are held at the same thermode temperature, differing by immediately preceding noxious thermal temperatures (**B**). In this subject, despite the same thermode temperature the reported pain intensity is higher during the t3 period for the Inv stimulus than for the constant control thermal stimulus. Similarly, offset stimulus (**C**) and stepdown (**D**) data are plotted with constant control T1 data to illustrate differences in pain intensity. Pain intensity changes are quantified in two ways. The first is a comparison between stepped and control stimuli (dotted arrows labeled “*a*”). For example in **B**, the *a* arrow shows the difference between the local maximum of the COVAS pain curve during the Inv stimulus and the pain intensity at the same timepoint during the control T2 stimulus. The second is a comparison made within a given stimulus (“*b*” arrows depict differences within the stepped stimuli between local maxima and minima of the COVAS pain curves). Differences between stepped and control stimuli (*a* arrows) and within-stimulus changes during stepped stimuli (*b* arrows) were extracted for group-level analysis.

Subjects were seated in a comfortable office chair in front of a desk. All heat stimuli were applied with the Pathway NeuroSensory Analyzer (Medoc; Ramat Yishai, Israel) using an fMRI-compatible, 3x3 cm thermode (ATS model). Subjects reported heat pain threshold with a button press using the Pathway Patient Response Unit. Subjects reported pain intensity in real time using a Computerized Visual Analogue Scale (COVAS, Medoc) consisting of a 100-mm visual analogue scale anchored by “no pain sensation” on the left and “most intense pain sensation imaginable” on the right (Fig 1A, white line above black slider). Subjects positioned a slider on this scale to reflect their pain intensity rating in that moment. Slider position over time was recorded using Medoc software.

### Cutaneous heat stimulation

All heat stimuli were applied to the volar surface of the non-dominant forearm. Three locations were used for heat stimulation, rotating from proximal to distal back to proximal. The time between heat stimuli was at least 120 seconds. With site rotation, the time between stimulating the same area of skin was at least 6 minutes. The thermode was placed on the volar forearm to allow full contact with the skin without excessive pressure and secured with a Velcro strap. The thermode was held at 32°C between heat stimuli.

### Heat pain threshold testing

Heat pain threshold was first determined using the method of limits. From a baseline of 32°C, the thermode was warmed at a rate of 1.5 C°/sec until subjects reported the transition from heat to pain via button press. The temperature reached was recorded as the heat pain threshold. The language used to describe the transition was “whenever the sensation changed from heat to pain.” Maximum cutoff temperature was set to 55°C. Heat pain thresholds from the three skin locations were averaged. This temperature was then used for heat stimulus calibration.

### Heat stimulus calibration

This portion of the protocol established an individually calibrated temperature to elicit a pain rating of ~50 mm on the COVAS at the end of a 30-second constant heat stimulus. The stimulus started at a baseline of 32°C, increased at a rate of 1.5 C°/sec, maintained a constant target temperature for 30 seconds, and returned to baseline temperature at a rate of 6 C°/sec. Subjects were asked to rate their pain on the COVAS in real time during each stimulus. The initial target temperature was chosen based on heat pain threshold. If the threshold was greater than or equal to 45°C, the initial target temperature was the heat threshold temperature. With lower thresholds, the initial target temperature was 2 C° higher than threshold. The stimulus target temperature was increased by 1 C° until pain report was between 40–60 mm on the COVAS. If pain report was greater than 90 mm, the subsequent stimulus was adjusted down by 1 C°. Finally, the temperature producing a pain report of ~50 mm on the COVAS was used for subsequent procedures and is referred to as T1. One C° higher was defined as T2.

### Supra-threshold heat pain testing

Subjects rated pain on the COVAS in response to a series of stepped and control supra-threshold stimuli (Fig 1A). Stepped stimuli were 30-second noxious heat stimuli featuring three time periods at different temperatures. The stepped stimuli were designed to investigate the impact of previous temperature steps on the final step period. The three step periods are referred to as t1 period, t2 period, and t3 period. Stepped stimuli had different temperature orders with the thermode held at the individually calibrated temperatures T1 or T2. The critical protocol used was the Inverted (Inv) stimulus which started at a baseline of 32°C, maintained at T2 for 5 seconds (t1 period), decreased by 1 C° to T1 for 5 seconds (t2 period), increased by 1 C° (back to T2) for 20 seconds (t3 period), and returned to baseline (Fig 1A & 1B). Initial temperature rise rate from baseline was 1.5 C°/sec. Rates of change after achieving T2 were 6 C°/sec. The stepped stimulus typically used to measure offset analgesia was included (offset stimulus, OS; rise rate 1.5 C°/sec, T1 for 5 seconds (t1 period), T2 for 5 seconds (t2 period), T1 for 20 seconds (t3 period); rates of change and fall rate 6 C°/sec; Fig 1A & 1C) as well as a novel stepdown stimulus (Sdn; rise rate 1.5 C°/sec, 10 seconds at T2 (t1 and t2 periods), 20 seconds at T1 (t3 period), rates of change and fall rate 6 C°/sec; Fig 1A & 1D). Constant control stimuli included a simple step to T1 (T1; 30 seconds at T1 (t1-3 periods), rise rate 1.5 C°/sec, fall rate 6 C°/sec; Fig 1A, 1C and 1D) and a simple step to T2 (T2; 30 seconds at T2 (t1-3 periods), rise rate 1.5 C°/sec, fall rate 6 C°/sec; Fig 1A & 1B).

The parameters for the offset stimulus (OS) and constant stimuli are similar to previously published reports investigating offset analgesia [7–9, 25–27]. The Inv and stepdown (Sdn) stimuli patterns are novel stimulus protocols with timing parameters and temperatures mirroring those of the OS stimulus. Temperature order of the Inv stimulus was manipulated to test whether an increase in temperature after a decrement produced a disproportionate increase in reported pain intensity. The Sdn stimulus is a modification of the OS stimulus to test whether eliminating the initial period at T1 affected offset analgesia magnitude. OS was repeated in triplicate. T1, T2, and Inv stimuli were repeated in duplicate. One Sdn was used. The number of replicates was chosen during preliminary testing. As noted above, the heat stimulus was sequentially rotated between three sites of the forearm. In pilot studies, three stimuli at each site (i.e. three rounds of three stimuli = 9 stimuli) was found to be feasible and acceptable to subjects (data not included). A T1 stimulus replicate was removed from the 9 stimuli and replaced with a Sdn stimulus. During data analysis, data from the T1 stimulus during the heat calibration procedure was included as a T1 replicate, allowing for the T1 condition to be considered in duplicate. The order of heat stimuli was randomized without replacement to ensure replicate testing using the randomization function in Excel (Microsoft, Redmond, Washington).

### Statistical analysis

Replicate pain intensity curves were averaged within each subject, and the resulting single mean values were used in all subsequent analyses. Since T1 was individually calibrated, pain intensity, thermode temperature, and time for each stimulus was sampled only during the timepoints at which thermode temperature was at T1 or greater (defined by T1-0.2°C). In each trial, this epoch was divided into t1 (5 seconds), t2 (5 seconds), and t3 (20 seconds) periods as described above and illustrated in Fig 1. Timeseries pain intensity data and pain intensity values at local extrema were used to quantify differences between stimulus protocols as follows.

For group-level analysis of pain intensity data over time, pain intensity and thermode temperature were aligned so that the initial timepoint for all subjects occurred at the first timepoint where thermode temperature was greater than T1-0.2°C. Data for replicate stimuli were averaged to obtain a subject-level mean at each timepoint. The subject-level mean was then downsampled to approximately 1.25 Hz. Group mean and 95% confidence intervals were then calculated for each timepoint. To compare the pain intensity timeseries data across stimuli, repeated measures 2-way ANOVAs were performed during the relevant time period with matching by stimulus, accounting for repeated measures within a subject, and time with post-hoc testing using Sidak’s multiple comparisons test.

To quantify perceptual differences between stimuli using previously described methods that yield a single value rather than timeseries data [27], extrema of pain intensity curves were extracted as follows: First, the timepoint of the t2 to t3 period transition was calculated using temperature data for each curve within each subject. The maximum or minimum, depending on the stimulus protocol, during a 10-second epoch centered on the t2-to-t3 transition was calculated. Then, the subsequent minimum or maximum was determined following the initially calculated extremum. This yielded a local extremum within the t3 period, graphically represented in Fig 1B–1D. For example, for the Inv stimulus pain intensity data, the minimum during the 10-second epoch centered on the t2-to-t3 transition was obtained. The maximum following this minimum was subsequently obtained and recorded (“MAX” in Fig 1B, “local maximum” in Fig 3A). Similarly, in the analysis of the Sdn pain intensity data, the maximum during the 10-second epoch centered on the t2-to-t3 transition was first obtained, followed by measurement of the minimum value following this maximum (“MIN” in Fig 1C, “local minimum” in Fig 3E). This method was used to extract local pain intensity extrema during all stimuli, including during the subtracted pain intensity curves (Figs 5 and 6).

For group-level analysis of differences across stimulus protocols, extrema were obtained from stepped curves within each subject as detailed above and then the group-level mean and 95% confidence intervals were calculated for each stepped protocol. For constant control stimuli (T1 and T2), the timepoint of the extreme value from the stepped stimulus data was used to extract COVAS pain intensity at the equivalent timepoint in the constant control stimulus data (Fig 1B–1D, arrow “a”).

For group-level analysis of within-stimulus change, extrema were obtained as detailed above and then subtracted. For example for Inv, the local minimum was subtracted from the local maximum (Fig 1B, “b” arrow). The value of the subtraction was then averaged across the group to obtain the group-level mean. The change in pain intensity during the same timepoints as the stepped pain intensity extrema was also calculated from the appropriate constant control pain intensity curves. For example, when comparing with the Inv stimulus pain intensity, pain intensity values from the T2 stimulus were obtained at the timepoints of the Inv pain intensity maximum and minimum. In this particular example, the direction of change for this measure during the T2 stimulus is negative, illustrated in Fig 3B.

A similar approach was applied to extract extrema and determine within-curve changes for the difference curves, which were calculated by simple subtraction of OS—T1 and Inv—T2. Again, these values were calculated within each subject, and then averaged for a group mean value.

For tests of statistical significance of single values per stimulus protocol per subject, paired t-tests were performed on subject-level mean values. Given the randomization of stimulus order, these values were treated as independent observations, and therefore did not require additional nested analyses. Univariate correlations were assessed by calculating Pearson’s r coefficient and accompanying p-values. 95% CI of Pearson’s r were calculated with Fisher’s transformation. Univariate linear regressions were calculated to draw regression lines in Fig 8.

Subject data were collected and managed using REDCap hosted at UCSF [28]. Anonymized data from REDCap were exported as a flat file and combined with anonymized sensory testing data and survey data using Excel (Microsoft, Redmond, Washington). Graphical and statistical analysis was performed using Matlab R2016b (The MathWorks, Natick, Massachusetts), StataMP v14 (Statacorp, College Station, Texas) and Prism 7 (GraphPad Software, La Jolla, California).

## Results

### A supra-threshold noxious heat stimulus is reported as more painful when immediately preceded by a rise in noxious thermal stimulus intensity

Healthy subjects (N = 74; 35 female and 39 male, mean age of 28.2 years ± SD 7.2 years with range 18–50 years) underwent heat pain testing using a 30 mm by 30 mm thermode applied to the volar surface of the non-dominant forearm (Fig 1). The group mean heat pain threshold was 44.6°C ± SD 1.7°C. Ascending noxious-range 30-second heat steps were then used to calibrate the specific temperatures used in suprathreshold heat stimuli for each subject. The temperature that elicited a pain intensity rating of 50 mm at the end of a 30-second stimulus was defined as the T1 temperature for an individual subject. The mean T1 used was 45.8°C ± SD 1.7°C ranging from 39°C to 47°C. T2 was defined as 1 C° higher than T1. Using individualized T1 and T2 values, subjects underwent a battery of randomly-ordered suprathreshold heat stimuli, including both stepped (Inv, OS, and Sdn) and constant control (T1 and T2) stimuli as outlined in Fig 1.

**Fig 2:**
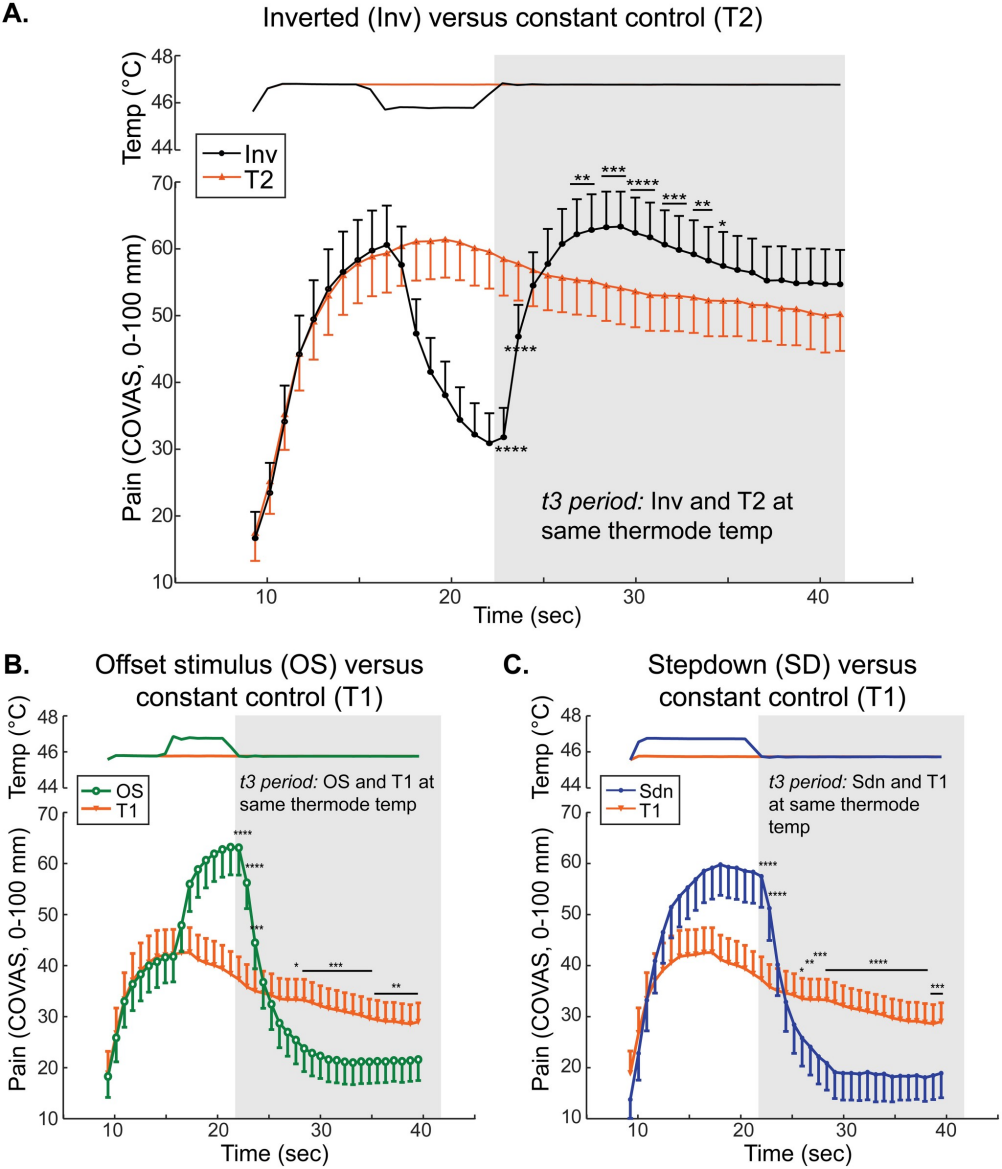
Pain intensity disproportionately increases with an increase in noxious-range temperature. **A.** Group mean temperature (top) and continuous pain intensity rating (bottom) curves from the Inverted (Inv, black circles) and T2 constant control (orange triangles) stimuli are shown. **B.** Group mean temperature (top) and continuous pain intensity rating (bottom) curves from the offset stimulus (OS, green circles) and T1 constant control (orange triangles) stimuli are shown. **C.** Group mean temperature (top) and continuous pain intensity rating (bottom) curves from the Stepdown (Sdn, blue circles) and T1 control (orange triangles) stimuli are shown. **A–C.** Symbols represent group-level means and error bars represent 95% confidence intervals. P-values: * p<0.05, ** p<0.01, *** p<0.001, **** p<0.0001.

We tested the hypothesis that a rising cutaneous noxious heat stimulus produces a greater reported pain intensity than a constant stimulus of the same intensity. Pain intensity ratings obtained during a novel stepped suprathreshold heat stimulus, Inv, and a constant control stimulus, T2, were compared (Fig 2A). During the t3 period in which thermode temperature is the same in both the Inv and control stimuli (shaded area in Fig 2A), reported pain intensity appeared greater during the Inv stimulus than during the constant control stimulus. To understand the effect of stimulus type (Inv vs. constant) on reported pain intensity over time, a 2-way repeated-measures ANOVA during the t3 period with matching by stimulus and time was calculated and demonstrated a significant main effect of stimulus type (F (1.000, 73.00) = 6.041, p = 0.0164), time (F (2.389, 174.4) = 23.27, p<0.0001), and time x stimulus type interaction (F (3.870, 282.5) = 46.54, p<0.0001). Sidak’s multiple comparison test confirmed statistically significant differences between Inv and constant control stimuli at each timepoint noted in Fig 2A with asterisks reflecting corrected p-value thresholds. During the t3 period, despite identical thermode temperatures, reported pain intensity following a noxious stimulus increase in the Inv stimulus was significantly higher than the reported pain intensity in the constant control stimulus.

### The offset of supra-threshold noxious heat disproportionately decreases pain intensity report with or without a preceding transient increase in noxious heat

To confirm the finding that small reductions in cutaneous noxious heat lead to disproportionate decreases in pain perception [8], pain intensity ratings during the stepped suprathreshold heat stimulus frequently used to elicit offset analgesia (offset stimulus, OS) were compared with pain intensity ratings during the constant control T1 stimulus (Fig 2B). Using pain intensity data from the t3 period of OS and constant control stimuli, a 2-way repeated-measures ANOVA with matching by stimulus and time demonstrated a significant main effect of stimulus type (F (1.000, 73.00) = 5.389, p = 0.0231), time (F (1.899, 138.6) = 90.93, p<0.0001), and time x stimulus type interaction (F (2.718, 198.4) = 88.62, p<0.0001). Sidak’s multiple comparison test confirmed statistically significant differences noted in Fig 2B with asterisks representing corrected p-value thresholds for between stimulus comparisons at each timepoint.

To test whether the preceding transient increase to T2 temperature is required for decreased pain intensity in the t3 period following a temperature offset, a stepdown design was used (Sdn stimulus). Pain intensities during the Sdn heat stimulus were compared with those during the constant control T1 stimulus (Fig 2C). During the t3 period in which thermode temperature is the same, pain intensity during the Sdn stimulus appears lower than the pain intensity during the constant control stimulus. This is a statistically significant difference, since a 2-way repeated-measures ANOVA with matching by stimulus and time showed a significant main effect of stimulus type (F (1.000, 73.00) = 15.16, p = 0.0002), time (F (2.122, 154.9) = 62.01, p<0.0001), and time x stimulus type interaction (F (3.171, 231.5) = 55.36, p<0.0001) with significant *post hoc* testing using the Sidak multiple comparison test (corrected p-values denoted in Fig 2C). Comparing pain intensity reported during the t3 period of either OS or Sdn with pain intensity during the same time period of the constant control stimulus demonstrates that a small decrease in noxious range temperature significantly decreases the reported pain intensity of a subsequent noxious thermal stimulus.

### Analysis of pain intensity extrema confirms that reported pain intensity is amplified or inhibited by supra-threshold changes in noxious stimulus intensity relative to constant control stimuli

To further characterize pain intensity differences between stepped and control stimuli, local extrema were extracted from pain intensity timeseries data during stepped stimuli (Inv, OS, and Sdn) as discussed in the Methods section and Fig 1. For each planned comparison, the pain intensity at the same timepoint was extracted from the corresponding constant control curve (T1 or T2). Within-subject extrema and constant control pain intensity values were then averaged across the group to allow for group-level comparisons. Additionally, within-stimulus pain intensity changes were calculated by subtracting the preceding minimum (for Inv) or maximum (for OS and Sdn) from the local extreme value in the t3 period (maximum for Inv and minimum for OS and Sdn; see Fig 1, arrow “b”). Similar between-stimuli and within-stimuli analyses have previously been used in studies of offset analgesia (e.g. [7, 9, 12, 25, 29, 30]).

**Fig 3:**
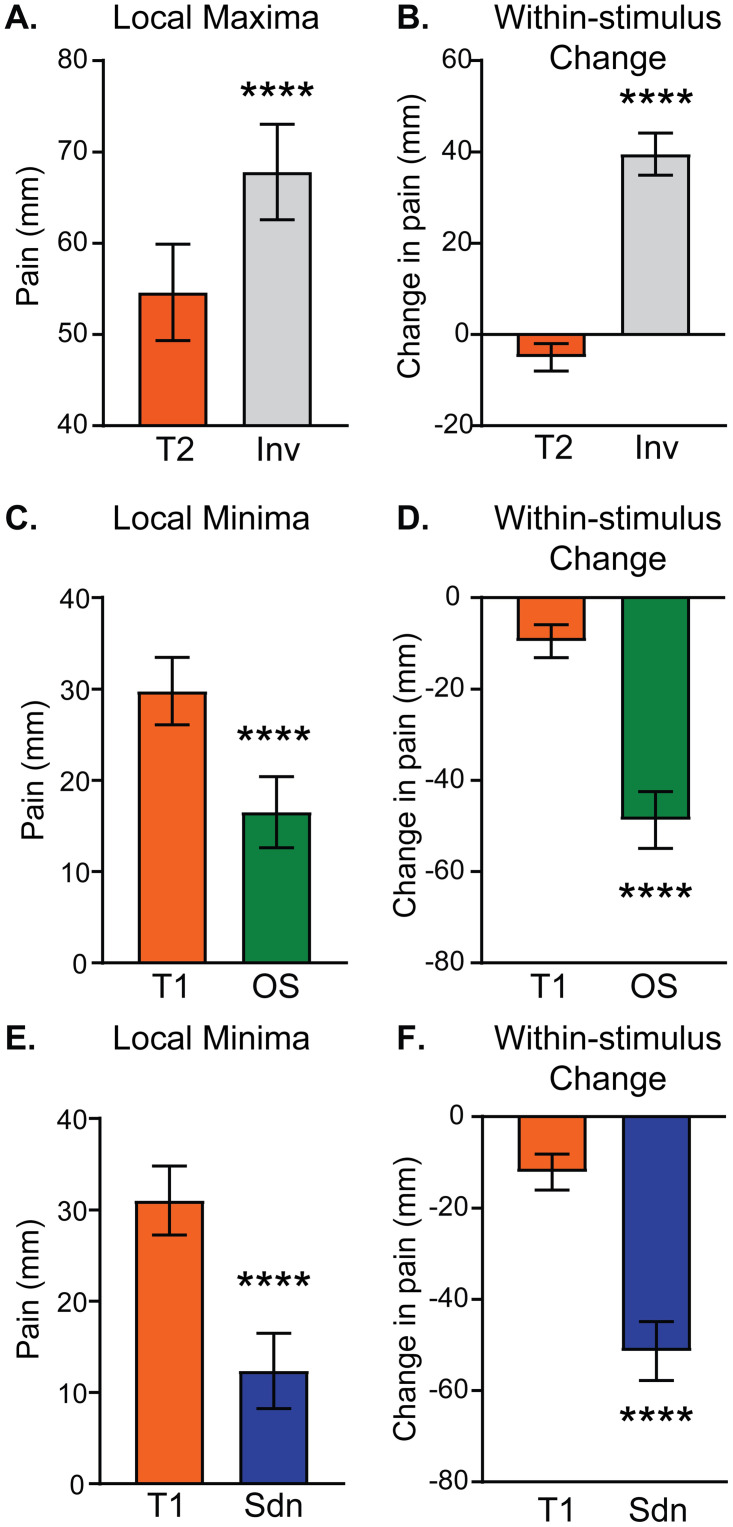
Extrema in continuously-rated pain intensity during stepped stimuli are significantly different than constant control stimuli. Pain intensity values on the computerized visual analogue scale were extracted at local maxima (A & B) and minima (C-F) for each subject as described in Fig 1. Pain intensities at matching timepoints were also extracted during constant control stimuli (T1 or T2). In the left column, pain intensities are compared between stepped and control stimuli (arrow “a” in Fig 1). In the right column, the change in pain intensity within the stepped curve is compared with the change in the control curve during the same time interval (arrow “b” in Fig 1). Group means with 95% CI are depicted in the bar graphs. Paired t-tests showed significant differences: **** p<0.0001.

Both analytic methods confirm that stepped stimuli, with preceding changes in supra-threshold noxious heat, elicit significantly different subjective pain intensities than constant control stimuli. The local maximum of reported pain intensity during the Inv stimulus is significantly greater than pain intensity during the constant control T2 stimulus at the equivalent timepoint (Fig 3A). Additionally, the within-stimulus pain intensity change (Fig 1B, arrow “b”) is significantly greater during the Inv stimulus than the change measured at equivalent timepoints during the constant control T2 stimulus (Fig 3B). Notably, the direction of change is different between the Inv and constant T2 stimuli. The constant T2 stimulus produces a decreasing pain intensity during the t3 period. Importantly, the pain intensity difference at the local maximum of the Inv stimulus (Fig 3A) is 13.2 mm (95% CI 9.3 mm– 17 mm), which, while opposite in sign, appears to be greater in absolute magnitude than an estimate of offset analgesia magnitude calculated in a recent quantitative meta-analysis of -4.6 mm (95% CI -7.5 mm–-1.7 mm) [27].

For noxious heat decreases, the pain intensity minima during both OS and Sdn stimuli (Fig 3C and 3E respectively) were significantly lower than equivalent timepoints (uniquely determined for OS and Sdn) during the control T1 stimulus. Similarly, the change in pain intensity within each stimulus was more negative, reflecting decreasing pain intensity, than that observed in the control T1 stimulus (Fig 3D and 3F).

### Despite producing an opposite direction of change, onset (Inv) and offset stimuli produce a similar absolute magnitude of change in subjective pain intensity

**Fig 4:**
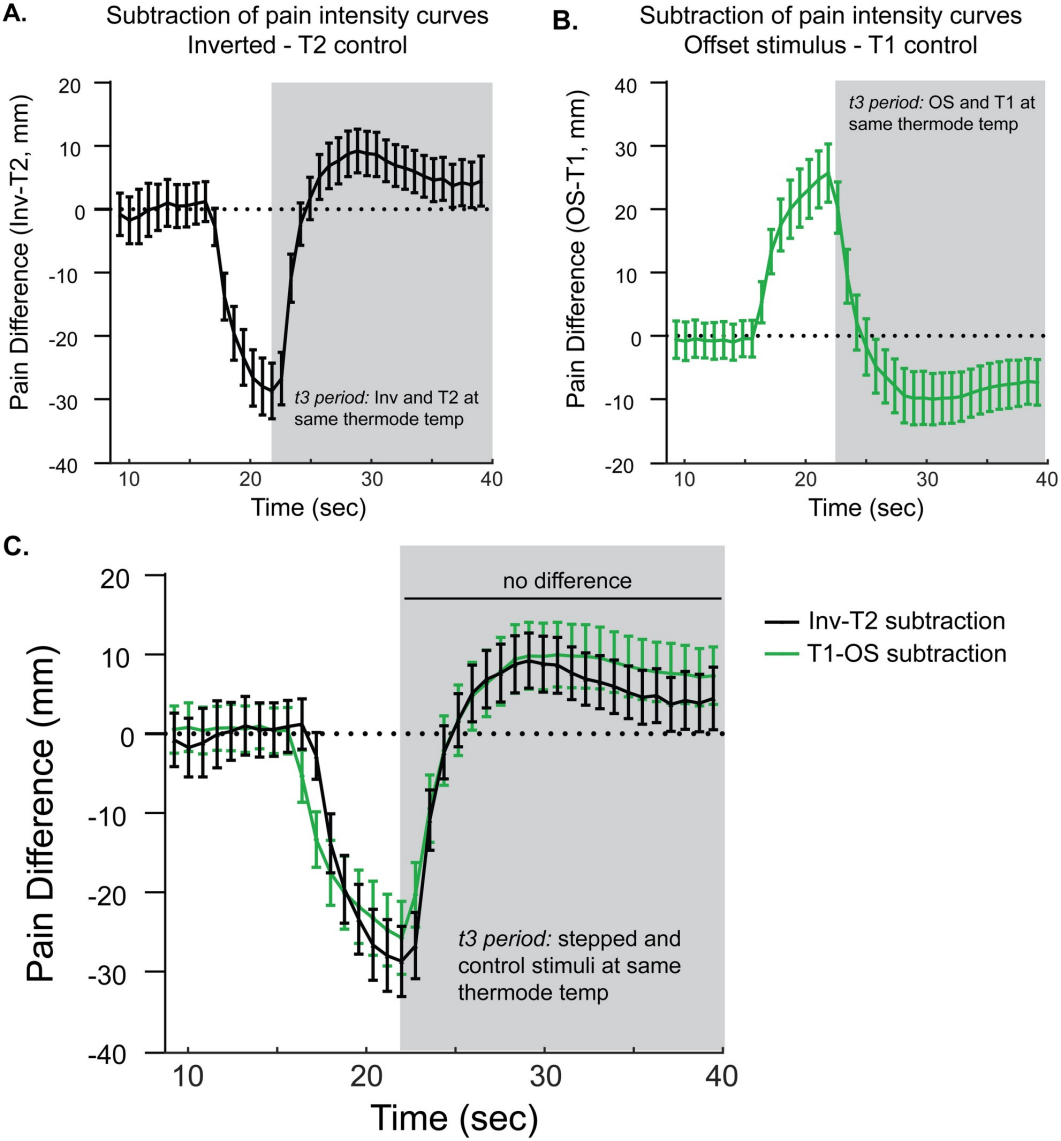
The absolute magnitude of relative hyperalgesia and hypoalgesia following equivalent temperature increases and decreases is similar. **A.** Pain intensity curves during Inv and T2 stimuli were subtracted for each subject with group mean values plotted; error bars = 95% CI. **B.** Similarly, pain intensity curves during OS and T1 stimuli were subtracted with group mean values plotted; error bars = 95% CI. In A. and B., shaded regions reflect the time period in which thermode temperatures are the same between stepped (Inv or OS) and constant (T2 or T1) stimuli. **C.** To compare pain difference curves, the inverse of the OS-T1 subtraction curve in B. was plotted with the Inv-T2 subtraction curve. Means and 95% CI are shown at each timepoint.

To account for time-dependent changes in pain intensity, such as adaptation, and allow for comparison across stepped stimuli, pain intensity curves during constant control stimuli, T2 and T1, were subtracted from pain intensity curves during stepped stimuli, Inverted (Inv) and Offset (OS) respectively. This was done within each subject and then averaged at each timepoint across the group. The Inv-T2 pain difference curve with 95% confidence intervals is plotted in Fig 4A, and the OS-T1 pain difference curve is plotted in Fig 4B. To compare their relative absolute magnitude, the OS-T1 pain difference was inverted and plotted on the same axis as the Inv-T2 pain difference curve (Fig 4C). Graphically, there appears to be substantial overlap between these two curves. During the t3 period, a repeated measures 2-way ANOVA with matching by both factors (subtraction curve and time) only showed a main effect of time (F (2.985, 217.9) = 83.84, p<0.0001) but no effect of subtraction curve type (F (1.000, 73.00) = 0.8225, p = 0.367) or time by curve type interaction (F (3.785, 276.3) = 2.440, p = 0.051). Post-hoc testing showed no significant differences between the Inv-T2 and T1-OS subtraction curves at individual timepoints (Sidak’s MCT). Overall, these results suggest that increases and decreases in temperature at the t2-to-t3 transition produce changes in pain intensity of opposite sign but highly similar absolute magnitude.

**Fig 5:**
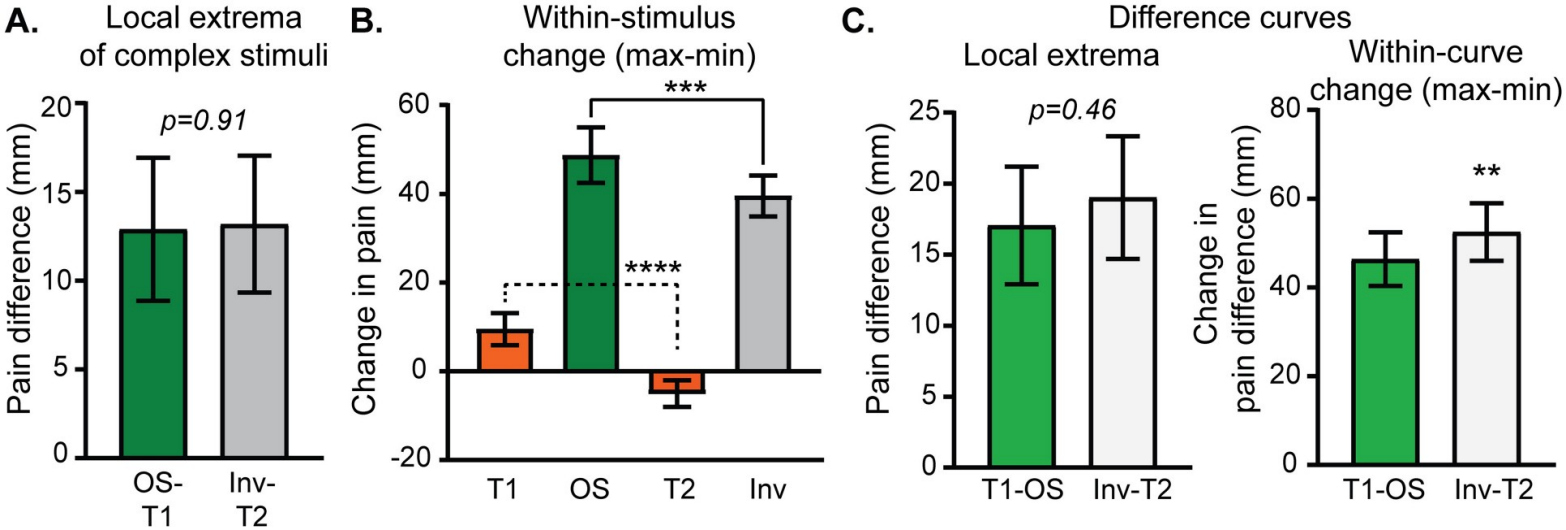
When controlled for adaptation by accounting for pain intensity changes during constant control stimuli, despite opposite directions of change, there is no significant difference in the change in absolute magnitude of pain report produced by stepped stimuli. **A.** The difference between pain intensity extrema (max for Inv, min for OS) and the pain intensity during control stimuli (T2 for Inv, T1 for OS), reflecting “a” arrows in Fig 1, was determined within each subject. **B.** Change in pain within stimulus (max—min, “b” arrows in Fig 1), for stepped stimuli and matched timepoints during control stimuli. A 1-way RM ANOVA showed significant main effect of stimulus. Post-hoc testing showed significant differences including those shown. **C.** Subtraction curves were analyzed within each subject for extrema and within-curve change. For all graphs, group mean with 95% CI error bars are plotted. P-values: ** p<0.01, *** p<0.001, **** p<0.0001.

Pain intensity reported during the stepped stimuli was further compared between Inv and OS stimuli using extrema and within-stimulus change analysis described above and in Fig 1. In Fig 5A, the absolute magnitude of the difference between stepped stimulus extrema (minimum for OS and maximum for Inv) and the equivalent timepoint in the constant control stimulus (T1 for OS and T2 for Inv; Fig 1, “a” arrows) was averaged across the group and plotted with 95% confidence intervals. A paired t-test shows no significant difference between these two values. In Fig 5B, the group mean average of within-stimulus change (Fig 1, “b” arrows), maximum—minimum for OS with equivalent timepoints during T1 and maximum—minimum for Inv and equivalent timepoints during T2, are plotted. To compare within-stimulus pain intensity change across stimulus type, a repeated measures 1-way ANOVA with matching by stimulus was calculated and showed a significant main effect of stimulus type (F (1.901, 138.8) = 149.7, p<0.0001). Planned post-hoc testing with Sidak’s MCT showed significant differences not only between OS and Inv, but also between the constant control stimuli (T1 versus T2). Interestingly, the mean differences between Inv and T2 (44.5 mm; 95% CI 36.9 mm-52.1 mm) and OS and T1 (39.2 mm; 95% CI 32.0 mm-46.4 mm) appeared similar, as did the mean differences between Inv and OS (9.2 mm; 95% CI 3.7 mm-14.6 mm) and T2 and T1 (14.5 mm; 95% CI 8.1 mm-21.0 mm). This suggests that the observed difference between Inv and OS in Fig 5B may actually be due to time-dependent changes shared across all stimuli (e.g. adaptation). To account for this possibility, the difference curves (Inv-T2 and T1-OS, plotted in Fig 5C) were analyzed. Within each subject, the maxima of the difference curves following the minima occurring around the t2-to-t3 transition was determined and then averaged across the group. Fig 5C (left) shows that the maxima of the Inv-T2 curve was no different than the maxima of the T1-OS (paired t-test; mean difference 1.9 mm; 95% CI -3.2 mm– 7.2 mm). The within-curve change of the subtraction curves was slightly greater in the Inv-T2 curve compared with the T1-OS curve (paired t-test; mean difference 6.1 mm; 95% CI 2.1 mm– 10.1 mm). Taken together, it appears that the increase in pain intensity following the noxious heat increase during the Inv stimulus is similar in magnitude to the decrease in pain intensity following the noxious heat decrease during the OS stimulus.

### A simple noxious stimulus intensity increase, if not preceded by an intensity decrement, produces a smaller hyperalgesic effect

**Fig 6:**
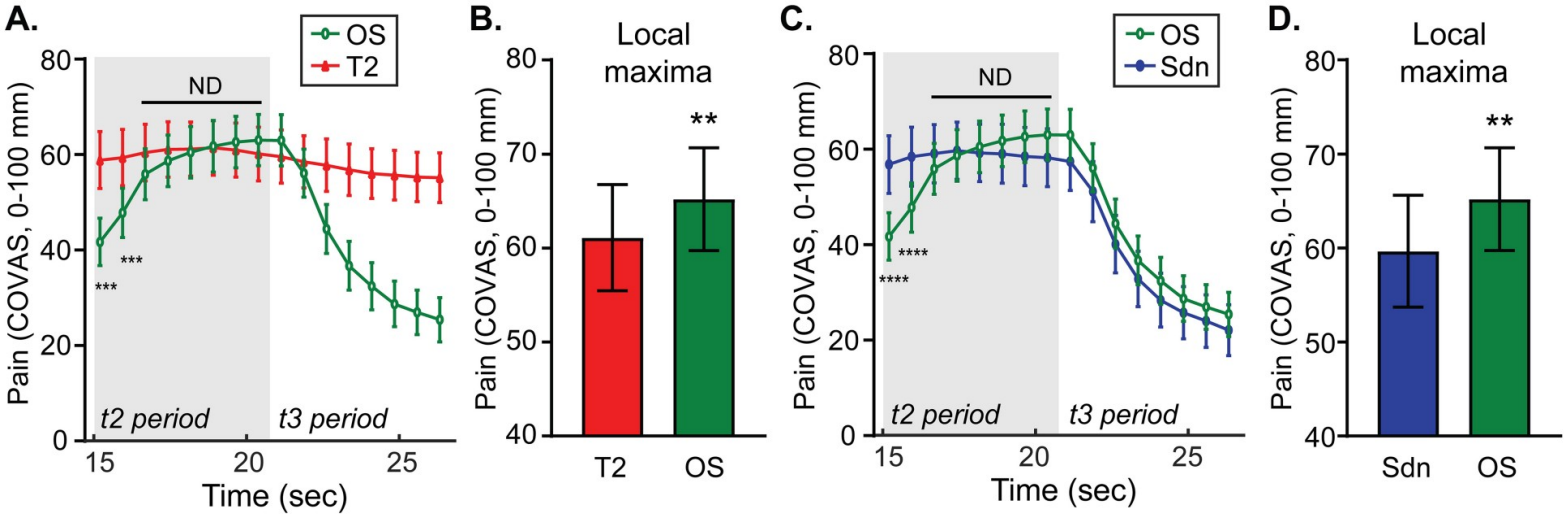
An increase in temperature within the noxious range without an immediately preceding decrease produces a comparatively small increase in pain intensity. **A.** Group mean continuous pain intensity rating curves during the t2 periods and beginning of the t3 periods of the OS (green circles) protocol and T2 control (red triangles) protocol are plotted. **B.** Group mean pain intensity local maxima during the OS stimulus protocol and the pain intensity at the equivalent timepoint during the control T2 stimulus are plotted. **C.** Group mean continuous pain intensity rating curves from the OS (green circles) and Sdn (blue circles) stimuli are plotted. **D.** Group mean pain intensity local maxima during the OS stimulus and the pain intensity at the equivalent timepoint during the control Sdn stimulus are plotted. For all graphs (A.–D.), group means are plotted with error bars representing 95% CI. P-values: ** p<0.01, *** p<0.001, **** p<0.0001. ND = no difference (p>0.05).

The effect of the temperature increase at the t1-to-t2 period transition on subsequent pain intensity was also explored. During the t2 period, the thermode temperatures are the same between offset, constant T2 and the stepdown stimulus protocols. Therefore, comparisons between protocols during the t2 period should inform the effect of a simple temperature increase. Group averaged time series data of pain intensity are plotted in Fig 6A and 6C. Group averaged local maxima during the OS stimulus are compared with pain intensity values during control stimuli, T2 (Fig 6B) and Sdn (Fig 6D). There were small differences between the OS and controls that reached statistical significance with paired t-tests. Repeated measures 2-way ANOVAs during the t2 period were calculated. For OS versus T2 (Fig 6A), there were main effects of time (F (1.634, 119.3) = 103.9, p<0.0001), stimulus (F (1.000, 73.00) = 6.801, p = 0.0110), and time x stimulus (F (1.790, 130.7) = 99.69, p<0.0001). For OS versus Sdn, there were main effects of time (F (1.422, 103.8) = 58.23, p<0.0001) and time x stimulus (F (1.594, 116.4) = 63.69, p<0.0001), but no main effect of stimulus (F (1.000, 73.00) = 1.364, p = 0.2466). Post-hoc testing using Sidak’s MCT showed significant differences labeled on the graphs with asterisks. Although apparent only with analysis of pain intensity curve maxima, there appeared to be a small increase in pain intensity during the t2 period of the offset stimulus (OS) protocol compared with control stimuli.

### A simple decrease in noxious heat elicits a subtly larger offset analgesia than a decrease preceded by an increase

**Fig 7:**
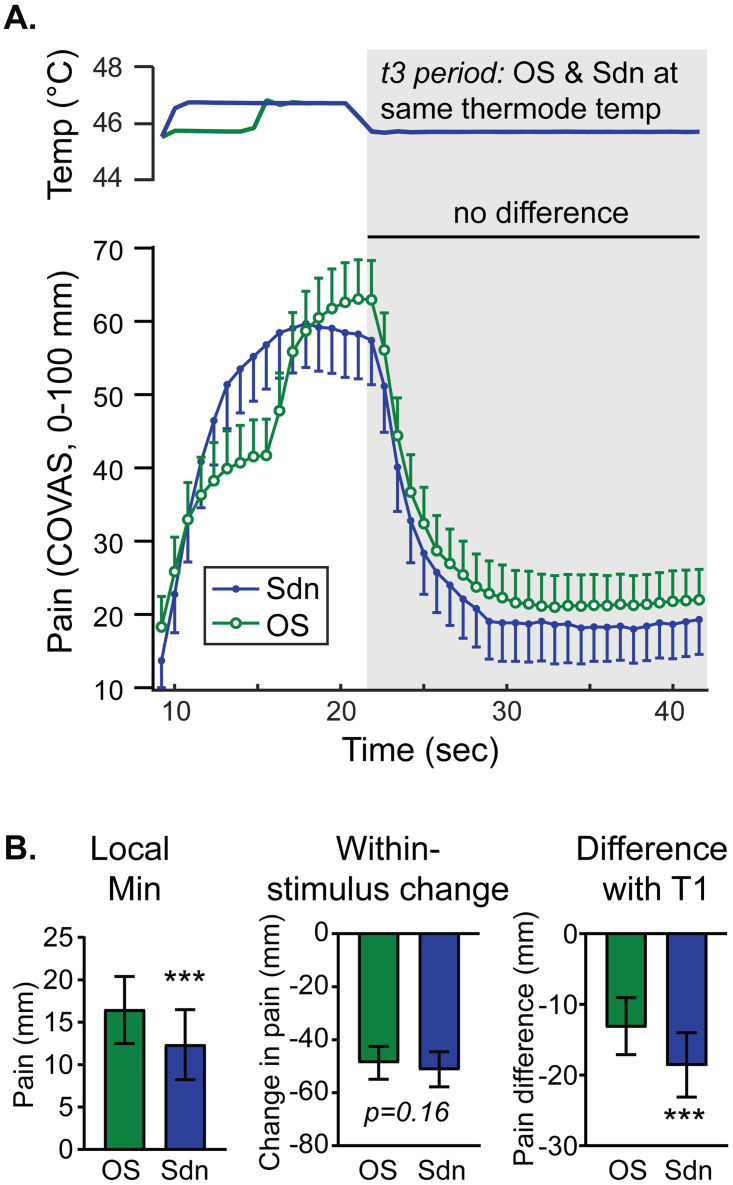
A noxious temperature decrease without a prior increase produces a subtly larger decrease in pain intensity. **A.** Group mean temperature (top) and continuous pain intensity rating (bottom) curves from the Sdn (blue circles) and OS (green circles) stimuli are shown. Symbols represent group-level mean and error bars represent 95% confidence intervals. Although a 2-way RM ANOVA with matching by stimulus and time showed main effects of time and stimulus, there was no statistically significant difference at any timepoint during the t3 interval. **B.** Group means obtained by within-subject analysis of minima (local min), the change in pain from maxima to minima (within-stimulus change) and the difference between stepped curve minima and T1 at equivalent timepoints (difference with T1) are plotted with error bars representing 95% CI. P-values: *** p<0.001.

Comparing pain intensity during the t3 periods of the offset and stepdown protocols interrogates the perceptual effect of a prior temperature increase on a subsequent decrease. Group mean timeseries data are plotted in Fig 7A with comparisons of local minima, within-stimulus change, and pain difference analyses shown in Fig 7B–7D. Comparing pain intensity at the local minima and the difference between the local minima the constant control stimulus T1 (Fig 1C & 1D, “a” arrows) shows a slightly larger magnitude offset analgesia (more negative) in the Sdn stimulus than the OS stimulus that reaches statistical significance (Fig 7B, paired t-tests). Fig 7B shows there is no difference when comparing within-stimulus changes in pain intensity between Sdn and OS stimulus protocols (Fig 1C & 1D, “b” arrows). A repeated measures 2-way ANOVA comparing pain intensity curves during the t3 period of Sdn and OS stimuli (Fig 7) revealed a main effect of time (F (2.325, 169.7) = 102.8, p<0.0001) and stimulus type (F (1.000, 73.00) = 8.518, p = 0.0047) without an interaction (F (3.852, 281.2) = 0.4906, p = 0.7357). Post-hoc testing with Sidak’s MCT showed no single timepoint difference achieved statistical significance. Taken together, it appears that offset analgesia during the t3 period is slightly larger in the stepdown stimulus compared with the offset stimulus.

### Although stimulus temperature is not correlated with offset analgesia or onset hyperalgesia, the magnitudes of the two are inversely correlated

**Fig 8:**
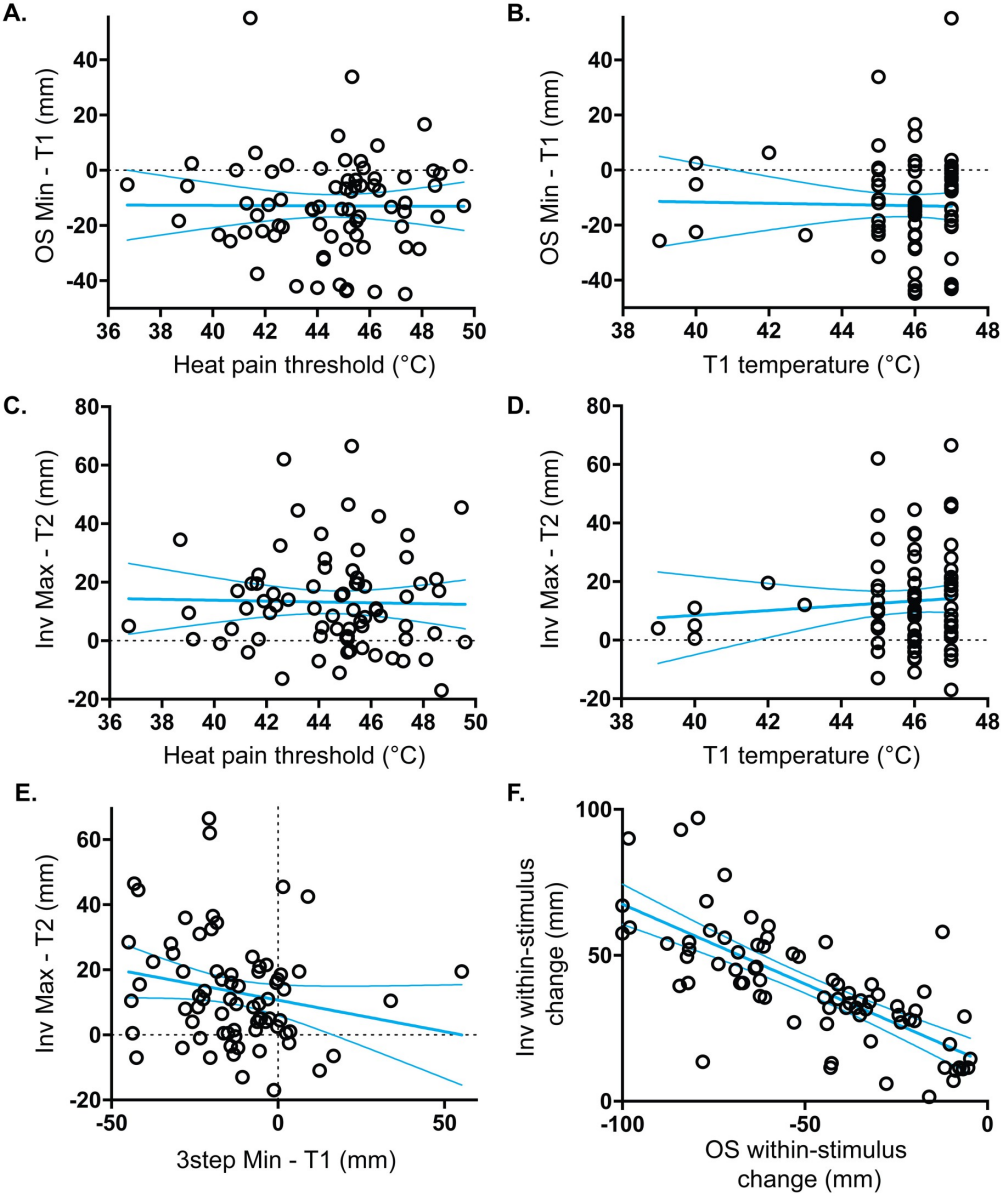
Pain intensity amplification with either increases or decreases are independent of temperature but are correlated with each other. Scatter plots are shown with each point representing an individual subject. Heavy cyan lines represent best-fit lines from linear regression analysis with thin cyan lines representing bounds of the 95% confidence intervals calculated as part of the linear regression. No correlation exists between stimulus intensity (heat pain threshold (A or C) or T1 stimulus temperature used (B or D)) and pain intensity modulation during the t3 period by either preceding noxious temperature decreases (OS Min-T1) or increases (Inv Max-T2). Although an inverse correlation between onset hyperalgesia and offset analgesia does not achieve statistical significance when comparing local extrema (E), a significant inverse correlation is detected using within-stimulus change (F).

In the initial characterization of offset analgesia in 12 volunteers, the magnitude of offset analgesia was consistent across a range of noxious temperatures [8]. In the current larger dataset, there again appears to be no correlation between temperature and magnitude of offset analgesia (Fig 8A, Table 1). No correlation exists between offset analgesia (measured as the local minimum during the 3-step stimulus minus the pain rating during the constant T1 stimulus, Fig 1C “a” arrow) and either heat pain threshold or T1 temperature (Fig 8A & 8B; R^2^<0.0001 and R^2^ = 0.0005 respectively). Additionally, there is no correlation between pain intensity amplification during the Inv stimulus protocol (“onset hyperalgesia”, measured as the local maximum during the Inv stimulus minus the pain rating at the same time point during the constant T2 stimulus, Fig 1B “a” arrow) and either heat pain threshold or T1 temperature (Fig 8C & 8D, Table 1; R^2^ = 0.0006 and R^2^ = 0.007 respectively). Using other measures of onset hyperalgesia, including within-stimulus change (Fig 1B & 1C, “b” arrows), local extrema of subtraction curves (Fig 6C), and within-curve change of subtraction curves (Fig 6C), again there was generally no correlation with stimulus temperature or other variables listed above (Table 1). We did find a weak correlation between Inv within-stimulus change (Table 1; Fig 1B, “b” arrows) and both T1 temperature used (R^2^ = 0.0762) and heat pain threshold (R^2^ = 0.0538), but this was not seen with other measures of onset hyperalgesia. Taken together, it appears that there is minimal relationship between the magnitude of onset hyperalgesia or offset analgesia and the initial noxious stimulus intensity prior to onset or offset of noxious stimulus intensity.

**Table 1:** Correlations between different measures of onset hyperalgesia and offset analgesia.

	Inv local extrema (Inv Max—T2)	OS local extrema (OS Min—T1)	Inv within-stimulus change	OS within-stimulus change	InvT2 subtraction extrema	OST1 subtraction extrema	InvT2 within-sub-curve change	OST1 within-sub-curve change	T1 used
Inv local extrema	1								
OS local extrema	***-0*.*2077***	1							
Inv within-stim change	0.4361*	-0.3261*	1						
OS within-stim change	-0.4091*	0.5131*	*-0*.7324*	1					
InvT2 sub extrema					1				
OST1 sub extrema					*-0*.2448*	1			
InvT2 within-sub-curve change							1		
OST1 within-sub-curve change							*-0*.7968*	1	
T1 used	0.0853	-0.0133	0.2760*	-0.0257	-0.0192	0.0067	0.0531	-0.0995	1
Heat pain threshold	-0.0238	-0.0123	0.2319*	0.1271	-0.0175	-0.09	0.0011	0.0599	0.5926*

Pearson r values for each pair-wise comparison are shown. Bold italic font denotes comparisons between different measures of onset hyperalgesia (using the Inv stimulus protocol) and offset analgesia (using the OS protocol).

* p<0.05.

As an initial comparison between onset hyperalgesia and offset analgesia, pairwise correlations were made using measures outlined above. Interestingly, there were moderate to strong inverse correlations observed using measures of within-stimulus change (see Fig 1B & 1C, “b” arrows; Table 1: r = 0.73, 95% CI 0.61–0.82; Fig 8F: R^2^ = 0.53, deviation of slope from zero: p<0.0001) and the within-subtraction-curve change (Table 1: r = 0.80, 95% CI 0.70–0.87). A weak inverse correlation was observed using subtraction curve extrema (Table 1: r = 0.25, 95% CI 0.017–0.448). Using the difference between stepped stimuli extrema and control stimuli (Fig 1B & 1C, “a” arrows), there was no significant correlation between pain intensity modulation following increases and decreases (Table 1: r = -0.22, 95% CI -0.416–0.022; Fig 8E: R^2^ = 0.041, deviation of slope from zero: p = 0.084). Neither offset analgesia nor onset hyperalgesia correlated well with age, sex, body mass index, socioeconomic status, self-report measures of pain catastrophizing, depression, anxiety, or impulsivity (univariate correlations; S1 Table). There were a few weak correlations that did achieve statistical significance that are noted in S1 Table. Overall, there does appear to be an inverse correlation between offset analgesia and onset hyperalgesia.

## Discussion

The disproportionate drop in subjective pain sensation elicited by a decreasing noxious stimulus has been termed offset analgesia (OA) [7, 8]. Here, we demonstrate a disproportionate enhancement when the noxious stimulus is increasing (onset hyperalgesia, OH). Several lines of evidence support the existence of OH found in our study. First, using a novel noxious heat stimulus protocol in which a transient intensity decrease from target temperature is followed by a return to target (Inv stimulus), we observed significantly elevated pain intensity ratings compared with those reported in the constant control stimulus held at target temperature throughout the stimulus. This was apparent using group-mean time series data (Fig 2) as well as analysis of local maxima and change in pain (Fig 3A & 3B). Additionally, comparing stepped curves (Inv and OS) showed similar absolute magnitude of OH and OA (Fig 4). Two different analyses also showed high similarity between OH and OA time course and magnitude measured as both difference from stepped curve extrema and within-stimulus changes (Figs 4 & 5). Consistent with the known influence of prior pain experience on current pain intensity, we observe that the magnitude of both OH and OA is affected by the prior trajectory of the noxious stimulus (Figs 6 & 7). Finally, within subjects the magnitude of OA has a moderate inverse correlation with that of OH (Fig 8 & Table 1). Overall, these results are consistent with a unifying model of OA and OH in which the direction of change of noxious stimulus intensity strongly influences pain perception.

Prior studies support the notion of OH. Using radiant heat with an infrared laser, Morch and colleagues demonstrated that there is perceptual enhancement of temperature increases compared with a constant stimulus [15]. However, their model predicting pain intensity showed larger magnitude perceptual enhancement with temperature decreases than increases. The current study extends these findings by reporting the first evidence of OH using contact cutaneous noxious heat stimuli and by demonstrating similar absolute magnitudes of OA and OH. Additionally, our findings provide empirical support for a non-linear model of pain intensity that incorporates perceptual feedback proposed by Apkarian and colleagues [14]. This model predicted a rapid increase in pain intensity observed with the increase from T1 to T2 during the t2 period of an OS stimulus. The authors noted the change to be more subtle than the OA effect, but did not further quantify it. We quantified this small increase in pain intensity by comparing the t2 periods of the OS protocol (Fig 7) with the constant T2 control protocol. Importantly, our observation of different magnitudes of OH and OA depending on the prior trajectory of the heat stimulus is consistent with a perceptual feedback model incorporating immediately preceding change in noxious stimulus intensity as a critical variable that modulates subjective pain intensity elicited by a given stimulus.

We also extended our understanding of OA by showing that it is present to a similar if not larger magnitude following a single step noxious heat decrease (Sdn) than when measured during the classical OS protocol. Our result contrasts with that of Haggard and colleagues who reported no changes in pain intensity with a single step temperature decrease compared with a constant stimuli [31]. This difference may be due to the use of predetermined temperatures rather than the psychophysically calibrated stimulus temperatures used in the current study or the possible confound of concurrent mechanical stimulation with Haggard and colleagues’ thermode setup. Haggard and colleagues suggest an alternative reason, in which a certain duration in the noxious range is required to elicit OA. Kurata and colleagues reported greater magnitude OA with a longer t2 period duration at the higher noxious temperature (T2) in the classical three-step OS protocol [32]. Taken together with our observation of robust OA with a single step noxious heat decrease in the Sdn stimulus, we suggest Haggard and colleagues would have found OA and OH with longer-duration noxious stimulation prior to the simple temperature changes.

Although identifying neural mechanisms of OH is beyond the scope of the current study, we did find similarity with OA, which is thought to involve central processing based on several behavioral and neuroimaging experiments [9, 10, 14, 26, 33, 34]. Like OA [8], the target temperature of the stepped protocols used does not correlate well with the magnitude of OH (Fig 8 and Table 1). Heat pain threshold, which was measured prior to supra-threshold testing, also does not correlate well with either OA or OH. Interestingly, there may be an inverse correlation between OA and OH, although the strength of correlation depends on how each is measured. Within-stimulus change in pain intensity (Fig 1 “b” arrows) and within-curve change of the subtraction curves show moderate inverse correlations between OH and OA (Fig 8F and Table 1). Analysis of extrema (Fig 1 “a” arrows) are not significantly correlated between OH and OA (Fig 8E and Table 1). Interestingly, within-stimulus change measures show only weak-moderate correlation with extrema difference measures (Fig 1B “b” versus “a” arrows), consistent with divergent measurement effects observed in a recent meta-analysis of OA [27]. Given the stronger correlation between OA and OH using the within-stimulus change, it seems possible that studies only analyzing that measure may be capturing a composite outcome reflecting both OA and OH [8, 9, 13, 25, 33, 35–37]. Certainly, additional studies are required to clarify the mechanisms of OH and its relationship to OA in different populations.

Given our findings of OH and subtle differences in magnitude of both OH and OA depending on immediately prior noxious stimulus intensity, we favor an explanatory model similar to that proposed by Apkarian and colleagues [14] and outlined in [38] whereby changing noxious stimulus intensity impacts predictions of pain and pain relief which in turn modulates nociceptive transmission and pain perception bidirectionally. We observed that the magnitude of OH was larger during the t3 period of the Inv protocol than during the t2 period of the OS protocol. This could be due to differences in how predicted pain intensity changes, which will differ between these two protocols. According to our proposed model, the transient drop during the t2 period in the Inv stimulus would predict that subsequent stimulus intensity will decrease; this would reduce the incentive to engage in an action to terminate the noxious input. Compared to a steady noxious thermal stimulus, the rise back to that same level after the earlier decrease reverses the direction of the prediction, changing the motivational state from indifferent to strongly biased toward responding to the noxious stimulus. According to the Motivation-Decision model [39], this switch in optimal action selection will engage a top down modulatory circuit that amplifies pain if the decision is to respond to it and inhibits pain if the decision is to ignore the pain. In different behavioral paradigms, changed predictions/expectations about pain intensity can elicit either increases or decreases in reported pain [6, 40, 41].

On the other hand, the magnitude of OA was only subtly greater during the Sdn stimulus as compared to the OS stimulus. This could still be due to within-stimulus predictions, since the Sdn stimulus did not have an initial period at T1, but only included a temperature decrease from T2 to T1. It is possible that the difference in perceptual enhancement between the protocols is smaller than that observed between the Inv and OS protocols because the trajectory of pain intensity was generally the same—a noxious stimulus increase followed by a decrease. This combined model of OA and OH magnitude is supported by prior theoretical work [14] and by the observation that longer durations of the t2 period in the classical OS paradigm produce larger magnitude offset analgesia [32], since predictions can be time-dependent and continued pain predicts subsequent pain.

While the neurobiology of OH and OA is uncertain, given that OA is associated with BOLD activation in brainstem [10, 13] and deactivation in spinal cord [34], it is likely that descending pain modulatory circuits are engaged. The classical descending opioid activated pain modulatory circuit exerts bidirectional control via two classes of medullary neurons projecting to the spinal cord dorsal horn: pain inhibiting OFF cells and pain facilitating ON cells. We hypothesize that prediction of pain relief by a transient drop in stimulus intensity leads to an increase in OFF cell firing and inhibition of nociceptive input at the spinal level. Extending this to OH, we hypothesize that prediction of increasing pain by a transient rise in stimulus intensity leads to an increase in ON cell firing and facilitation of nociceptive input at the spinal level. Future work is needed to address this model [38].

Alternative explanations for our observation of OH remain possible. Although our study design controls for time-dependent within-stimulus changes in pain intensity, such as adaptation, by incorporating constant control stimuli (T1 and T2), it is possible that there are independent competing time-dependent processes producing the pain intensity curves. For example, the increase in pain intensity in the t3 period of the Inv stimulus may result from temporal summation which is distinct from pain inhibiting processes, such as adaptation or OA. We do not favor this explanation since heat pain temporal summation occurs with more frequent temperature changes of at least 0.33 Hz and not at 0.25 Hz or less [42], which is in the range of the Inv stimulus. Alternatively, the temperature decrement in the Inv stimulus during the t2 interval may reverse a single pain inhibiting process. The current study cannot rule this possibility out, but given the known bidirectional effects of expectancy/predictions on pain intensity and previous reports supportive of OH without the temperature decrement [14, 15], we favor the motivation-decision model. Future studies designed to manipulate pain and pain relief predictions will help delineate mechanisms on the behavioral level.

### In conclusion

The current study establishes the existence of OH and posits a unifying CNS-mediated model of OA and OH emphasizing how the predictive nature of changes in pain intensity strongly modulates individual responses to such changes through top down bidirectional modulation of nociceptive transmission.

## Supporting information

S1 Table Correlation coefficients between measures of onset hyperalgesia, offset analgesia, and psychosocial attributes. Pearson r values for each pair-wise comparison are shown. * p<0.05.
(DOCX)
