# Supplementary material for: Onset hyperalgesia and offset analgesia: Transient increases or decreases of noxious thermal stimulus intensity robustly modulate subsequent perceived pain intensity
Source: PLoS One. 2020 Dec 8;15(12):e0231124. doi: 10.1371/journal.pone.0231124 (PMC7723268; doi:10.1371/journal.pone.0231124)
Supplement: S1 Table — Pearson r values for each pair-wise comparison are shown. * p<0.05. (DOCX) [file pone.0231124.s001.docx]

**S1 Table: Correlation coefficients between onset hyperalgesia, offset analgesia, and psychosocial attributes.**

|  | Inv local extrema | OS local extrema | Inv within-stimulus change | OS within-stimulus change | InvT2 subtraction extrema | OST1 subtraction extrema | InvT2 within-sub-curve change | OST1 within-sub-curve change | T1 used | Heat pain threshold |
| --- | --- | --- | --- | --- | --- | --- | --- | --- | --- | --- |
| Pain catastrophizing (PCS) | 0.01 | 0.0321 | -0.003 | -0.0014 | 0.089 | 0.0129 | 0.0038 | 0.0016 | -0.0999 | 0.0022 |
| Depression (BDI-II) | 0.0833 | -0.1522 | -0.0554 | 0.0317 | -0.1227 | 0.0499 | -0.0686 | 0.0055 | 0.037 | 0.063 |
| Trait Anxiety (STAI-Y2) | -0.0437 | -0.0574 | -0.1114 | 0.0668 | -0.0272 | -0.0394 | -0.1564 | 0.0055 | 0.0116 | 0.071 |
| Impulsivity (BIS-11) | -0.0443 | -0.075 | 0.104 | 0.0403 | -0.012 | -0.0953 | -0.0544 | -0.0016 | 0.177 | 0.0621 |
| Situational pain catastrophizing (SPCS) | 0.07 | -0.1825 | 0.2093 | -0.2473* | -0.14 | 0.092 | 0.1593 | -0.2572* | 0.2493* | 0.0832 |
| State anxiety (STAI-Y1) | 0.134 | -0.1783 | 0.0599 | -0.1341 | -0.1882 | 0.1352 | 0.0213 | -0.1506 | 0.1032 | 0.2015 |
| State anxiety after testing (STAI-Y1) | 0.0735 | -0.0662 | 0.1421 | -0.1004 | -0.0842 | 0.0277 | 0.0158 | -0.0838 | 0.2239 | 0.1103 |
| Social status (BSMSS) | -0.0146 | -0.0299 | -0.0101 | 0.0656 | -0.0215 | -0.0284 | -0.0054 | 0.0465 | 0.0168 | -0.0466 |
| Age | -0.0712 | -0.0093 | 0.0768 | 0.1843 | -0.0148 | -0.1002 | -0.0539 | 0.161 | 0.0363 | 0.2340* |
| BMI | -0.1961 | 0.0285 | -0.1746 | 0.3276* | 0.0445 | -0.2373* | -0.2894* | 0.2656* | 0.1207 | 0.1379 |

Pearson r values for each pair-wise comparison are shown. * p<0.05.
